# Crystal structure of an organic–inorganic supra­molecular salt based on a 4,4′-methyl­enebis(3,5-dimethyl-1*H*-pyrazol-2-ium) cation and a β-octa­molybdate anion

**DOI:** 10.1107/S2056989015024524

**Published:** 2016-01-06

**Authors:** Tatiana R. Amarante, Isabel S. Gonçalves, Filipe A. Almeida Paz

**Affiliations:** a Department of Chemistry, CICECO – Aveiro Institute of Materials, University of Aveiro, 3810-193 Aveiro, Portugal

**Keywords:** crystal structure, 4,4′-methyl­enebis(3,5-dimethyl-1*H*-pyrazol-2-ium) cation, β-octa­molybdate anion, hydrogen-bonding network

## Abstract

The asymmetric unit of the title compound, bis­[4,4′-methyl­enebis(3,5-dimethyl-1*H*-pyrazol-2-ium)] β-octa­molybdate, (C_11_H_18_N_4_)_2_[Mo_8_O_26_] or (H_4_mbdpz)_2_[Mo_8_O_26_], is composed of an H_4_mbdpz^2+^ cation and half of the β-octa­molybdate anion which is completed by inversion symmetry. The organic mol­ecular units are engaged in a series of N—H⋯O hydrogen bonds with neighbouring anions, with N⋯O distances and N—H⋯O angles in the ranges 2.730 (2)–2.941 (2) Å and 122–166°, respectively. These inter­actions lead to the formation of a supra­molecular two-dimensional network parallel to the (010) plane.

## Abstract 2

The crystal structure of the title novel organic–inorganic supra­molecular salt is based in the *in situ* formation of 4,4′-methyl­enebis(3,5-dimethyl-1*H*-pyrazol-2-ium) cations, which are engaged in N—H⋯O hydrogen bonds with β-octa­molybdate anions.

## Chemical context   

4,4′-Methyl­enebis(3,5-di­methyl­pyrazole) (H_2_mbdpz) is a flexible organic mol­ecule which has been extensively used in the last few years by various research groups to design coordin­ation-based and organic solids. While, on the one hand, the central methyl­ene moiety confers some conformational flexibility to the entire mol­ecular unit, on the other the two peripheral pyrazole rings permit not only the coordination to various types of metal atoms but also the involvement of these moieties in complex networks based on hydrogen bonds. It is, thus, not surprising to encounter a rich chemistry and structural diversity associated with this mol­ecule. A search in the literature and in the Cambridge Structural Database (CSD; Allen, 2002 ▸; Groom & Allen, 2014 ▸) reveals, for example, that H_2_mbdpz has been used as an effective bending spacer to construct a large number of metal-organic frameworks (MOFs) or coordination polymers with various remarkable topologies based on a rather diverse range of *d*-block metals (Goswami *et al.*, 2013 ▸; Mondal *et al.*, 2008 ▸; Timokhin *et al.*, 2015 ▸). H_2_mbdpz and its derivatives have also been used to prepare a range of supra­molecular networks based on either neutral organic mol­ecules or in the formation of salts with a wide range of anions (since, typically, the two pyrazole moieties appear protonated) (Basu *et al.*, 2009 ▸; Basu & Mondal, 2010 ▸; Hazra *et al.*, 2010 ▸). Most of these structural reports available in the literature either use H_2_mbdpz purchased from commercial sources or the authors prepare the mol­ecule using published procedures. For the latter case, the standard method dates back to that reported by Trofimenko (1970 ▸), but more recent and alternative approaches are also employed to prepare the intended mol­ecule (Kruger *et al.*, 2000 ▸).
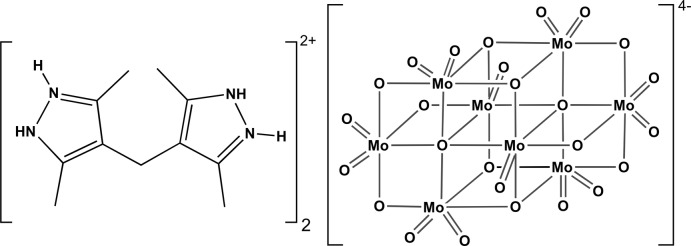


In this communication, we report the unexpected isolation of a new supra­molecular salt in which 4,4′-methyl­enebis(3,5-dimethyl-1*H*-pyrazol-2-ium) (H_4_mbdpz^2+^) is prepared *in situ*, inside the autoclave reaction vessel, starting from 3,5-di­methyl­pyrazole in a reaction catalysed by Mo^VI^ ions in the presence of hydrogen peroxide. To balance the cationic charge of the protonated H_4_mbdpz^2+^ moiety, the crystal contains the well-known β-octa­molybdate anion. It is remarkable to note that, despite the intensive research on supra­molecular structures based on H_2_mbdpz, only a couple of very recent reports contain polyoxidometalate-type anions. Indeed, Tian *et al.* (2014 ▸, 2015 ▸) described various Ag^+^-based MOFs (or coord­in­ation polymers) in which Mo^VI^ or W^VI^ Keggin and/or Wells–Dawson polyoxidometalates balance the positive charge of the cationic architectures.

## Structural commentary   

**Figure 1:**
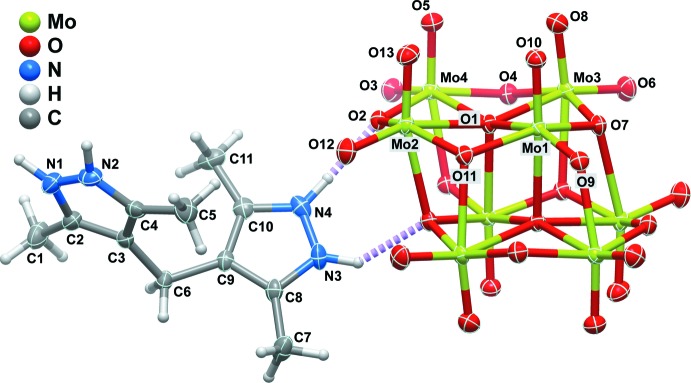
Schematic representation of the mol­ecular entities composing the asymmetric unit of the title compound. The β-octa­molybdate anion has been completed by inversion symmetry for the sake of chemical accuracy. All non-hydrogen atoms are represented as displacement ellipsoids drawn at the 60% probability level and hydrogen atoms as small spheres with arbitrary radii. Non-hydrogen atoms belonging to the asymmetric unit have been labelled for clarity. Dashed violet lines indicate N—H⋯O hydrogen-bonding inter­actions (see Table 1 ▸ for geometrical details).

The asymmetric unit of the title compound is composed of a 4,4′-methyl­enebis(3,5-dimethyl-1*H*-pyrazol-2-ium) cation (H_4_mbdpz^2+^), and one half of the β-octa­molybdate anion, β-[Mo_8_O_26_]^4−^ (Fig. 1 ▸).

The H_4_mbdpz^2+^ cation exhibits the typical structural features found in related compounds. The considerable steric hindrance imposed by the two peripheral 3,5-dimethyl-1*H*-pyrazol-2-ium moieties induces a tetra­hedral angle of the bridging methyl­ene group of 113.56 (17)°, which is very close to the median value found in similar structures (from the CSD: median of 114.7° from 109 hits with range of 111.0–120.0°). Conversely, the dihedral angle subtended by these two peripheral moieties is significantly more dependent on the crystal structure itself, with the literature values (from 109 hits in the CSD) ranging from as low as 55.1° (a chiral coordination polymer with Cu^2+^ described by Lin *et al.*, 2014 ▸) to 90.0° (an Ni^2+^ layered network described by Goswami *et al.*, 2013 ▸). Nevertheless, the inter­planar angle registered for the title compound, 77.85 (15)°, agrees well with the median value of all structures deposited in the CSD (81.1°).

The mol­ecular geometrical parameters for the β-octa­molybdate anion are typical, exhibiting the usual four families of Mo—O bonds: Mo—O*t* to terminal oxido groups [bond lengths in the 1.6883 (14)–1.7077 (15) Å range]; Mo—O*b* to μ_2_-bridging oxido groups [bond lengths in the 1.7506 (15)–2.2304 (15) Å range]; Mo—O*c* to μ_3_-bridging oxido groups [bond lengths in the 1.9431 (14)–2.4033 (14) Å range]; Mo—O*c* to μ_5_-bridging oxido groups [bond lengths in the 2.1441 (14)–2.3577 (14) Å range]. The four crystallographically independent Mo^VI^ metal atoms are hexa­coordinated in a typical {MoO_6_} fashion resembling highly distorted octa­hedra: while the *trans* inter­nal O—Mo—O octa­hedral angles are found in the 142.75 (6)–174.00 (6)° range, the *cis* angles refine instead in the 71.04 (5)–105.61 (8)° inter­val. This wide dispersion for the inter­nal octa­hedral angles is a notable and well-known consequence of the marked *trans* effect created by the terminal oxido groups, which displace the metal atoms from the center of the octa­hedra. The inter­metallic Mo^VI^ distances within the β-octa­molybdate anion range from 3.1875 (5) Å (for the Mo1⋯Mo2 distance) to 3.5810 (5) Å [for the Mo1⋯Mo1^i^ distance across the inversion center of the anion; symmetry operation: (i) −*x*, 1 − *y*, 1 − *z*].

## Supra­molecular features   

**Figure 2:**
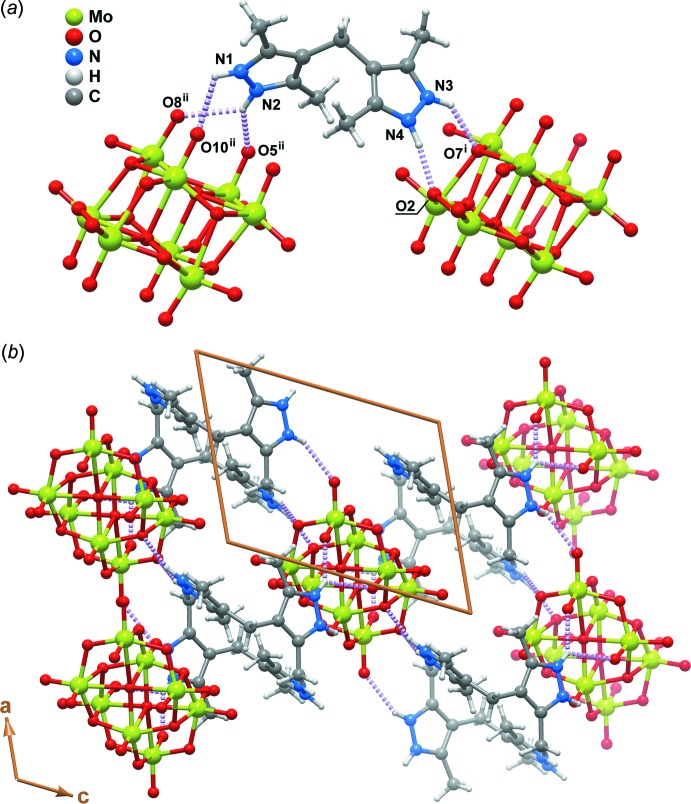
Schematic representation of the type and role of N—H⋯O hydrogen bond inter­actions present in the crystal structure of the title compound: (*a*) description of all inter­actions which connect the crystallographically independent 4,4′-methyl­enebis(3,5-dimethyl-1*H*-pyrazol-2-ium) cation to two neighbouring β-octa­molybdate anions; (*b*) portion of the two-dimensional supra­molecular layer placed in the *ac* plane of the unit cell formed by the connection between the mol­ecular units present in the title compound. For geometrical details of the represented hydrogen bonds (as violet dashed lines) see Table 1 ▸. Symmetry operations used to generate equivalent atoms: (i) −*x*, 1 − *y*, 1 − *z*; (ii) −*x*, 1 − *y*, −*z*.

The crystal packing of the title compound is essentially mediated by the presence of various N—H⋯O hydrogen-bonding inter­actions between the H_4_mbdpz^2+^ cation (which acts as the donor – *D*) and the β-octa­molybdate anion (the acceptor – *A*) (Fig. 2 ▸ *a*). As depicted in Table 1 ▸, the *D*⋯*A* distances are relatively short, ranging between 2.730 (2) and 2.977 (2) Å. It is noteworthy that the latter is associated with the N2—H2 group which is engaged in a bifurcated inter­action with the neighbouring β-octa­molybdate anion (as depicted in Fig. 2 ▸ *a*), hence leading to an average increase of the inter­atomic distances.

Besides these inter­actions, the crystal structure is also rich in weak hydrogen bonds of the C—H⋯O type (not shown) involving mainly the terminal methyl groups of the organic mol­ecule. The various C—H⋯O inter­actions present in the crystal structure are rather weak, with C⋯O distances ranging from 3.203 (3) to 3.457 (3) Å, with <(CHO) inter­action angles in the 123–168° inter­val.

**Figure 3:**
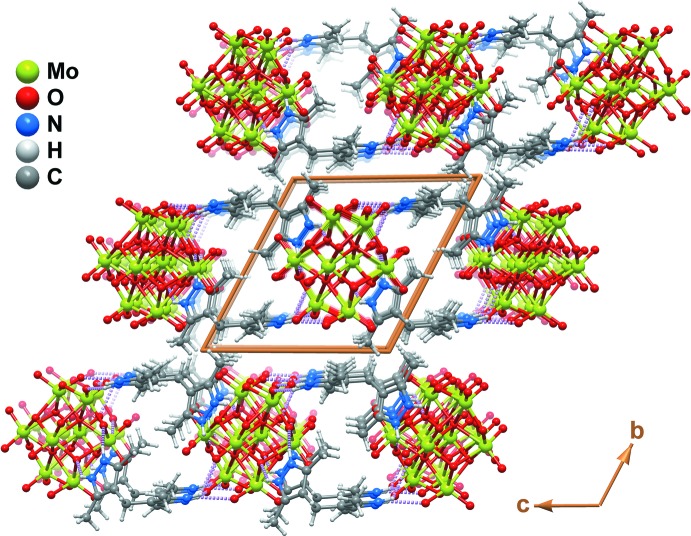
Ball-and-stick schematic representation of the crystal packing of the title compound viewed in perspective along the [100] direction. The figure emphasizes, on the one hand, how the inorganic component of the crystal structure is fully embedded into an organic matrix based on the 4,4′-methyl­enebis(3,5-dimethyl-1*H*-pyrazol-2-ium) cation. On the other it shows how supra­molecular hydrogen-bonded layers pack closely perpendicular to (010).

The aforementioned hydrogen bonds between cations and anions lead to the formation of a two-dimensional supra­molecular network parallel to the (010) plane (Fig. 2 ▸ *b*). Individual supra­molecular entities close-pack perpendicular to (010) to produce the crystal structure of the title compound (Fig. 3 ▸).

## Synthesis and crystallization   

MoO_3_ (Analar, BDH Chemicals, 99.5%), 3,5-di­methyl­pyrazole (Aldrich, 99%) and H_2_O_2_ (50% in water, Sigma–Aldrich) were obtained from commercial sources and used as received. FT–IR spectra were collected using KBr pellets (Sigma–Aldrich, 99%, FT–IR grade) on a Mattson-7000 infrared spectrophotometer.

A mixture of MoO_3_ (0.349 g, 2.42 mmol), 3,5-di­methyl­pyrazole (0.116 g, 1.21 mmol), water (23 mL) and H_2_O_2_ (2 mL) was heated in a Teflon-lined stainless steel digestion bomb at 433 K for 26 h, at 373 K for 25 h, and finally slowly cooled down to ambient temperature over a period of 13 h. Single crystals of the title compound were obtained inside the Teflon vessel along with a yellow aqueous mother liquor (pH = 6) and a blueish solid, which was confirmed by powder X-ray diffraction studies to be residues of unreacted MoO_3_.

FT–IR (cm^−1^): ν~ = 3218 (*vs*); 3127 (*s*); 3008 (*s*); 2859 (*s*); 2719 (*s*); 1606 (*m*); 1579 (*s*); 1535 (*m*); 1517 (*m*); 1438 (*s*); 1394 (*m*); 1365 (*m*); 1253 (*m*); 1184 (*m*); 1153 (*w*); 1070 (*w*); 1047 (*w*); 948 (*vs*); 925 (*s*); 908 (*vs*); 844 (*s*); 721 (*s*); 705 (*s*); 671 (*s*); 655 (*s*); 543 (*s*); 522 (*m*); 480 (*w*); 458 (*w*); 445 (*w*); 414 (*m*); 401 (*m*); 360 (*m*).

## Refinement details   

Crystal data, data collection and structure refinement details are summarized in Table 2 ▸. Hydrogen atoms bound to carbon were placed at idealized positions with C—H = 0.99 and 0.98 Å for the –CH_2_– and methyl groups, respectively, and included in the final structural model in the riding-motion approximation with isotropic displacement parameters fixed at 1.2 or 1.5*U*_eq_, respectively, of the carbon atom to which they are attached.

Hydrogen atoms associated with nitro­gen atoms were directly located from difference Fourier maps and included in the model with the N—H distances restrained to 0.95 (1) Å in order to ensure a chemically reasonable environment for these moieties. These hydrogen atoms were modelled with isotropic thermal displacement parameters fixed at 1.5*U*_eq_(N).

## Supplementary Material

Crystal structure: contains datablock(s) I, New_Global_Publ_Block. DOI: 10.1107/S2056989015024524/gk2651sup1.cif

Structure factors: contains datablock(s) I. DOI: 10.1107/S2056989015024524/gk2651Isup2.hkl

CCDC reference: 1443502

Additional supporting information:  crystallographic information; 3D view; checkCIF report

## Figures and Tables

**Table 1:** Hydrogen-bond geometry (Å, °)

*D*—H⋯*A*	*D*—H	H⋯*A*	*D*⋯*A*	*D*—H⋯*A*
N1—H1⋯O10^i^	0.94	2.34	2.941 (2)	122
N2—H2⋯O5^i^	0.94	2.05	2.852 (2)	143
N2—H2⋯O8^i^	0.94	2.31	2.977 (2)	127
N3—H3⋯O7^ii^	0.94	1.97	2.759 (2)	141
N4—H4⋯O2	0.94	1.81	2.730 (2)	166

Symmetry codes: (i) 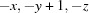; (ii) 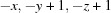.

**Table 2:** Experimental details

Crystal data	
Chemical formula	(C_11_H_18_N_4_)[Mo_8_O_26_]
*M*_r_	1596.11
Crystal system, space group	Triclinic, *P* 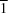
Temperature (K)	180
*a*, *b*, *c* (Å)	8.6394 (10), 12.0694 (13), 12.2249 (14)
α, β, γ (°)	113.343 (3), 110.629 (4), 96.540 (4)
*V* (Å^3^)	1046.6 (2)
*Z*	1
Radiation type	Mo *K*α
μ (mm^−1^)	2.42
Crystal size (mm)	0.28 × 0.18 × 0.15

Data collection	
Diffractometer	Bruker D8 QUEST
Absorption correction	Multi-scan (*SADABS*; Bruker, 2001 ▸)
*T*_min_, *T*_max_	0.595, 0.746
No. of measured, independent and observed [*I* > 2σ(*I*)] reflections	58761, 5605, 4669
*R*_int_	0.032
(sin θ/λ)_max_ (Å^−1^)	0.685

Refinement	
*R*[*F* ^2^ > 2σ(*F* ^2^)], *wR*(*F* ^2^), *S*	0.021, 0.045, 1.05
No. of reflections	5605
No. of parameters	305
No. of restraints	4
H-atom treatment	H atoms treated by a mixture of independent and constrained refinement
Δρ_max_, Δρ_min_ (e Å^−3^)	0.45, −0.38

Computer programs: *APEX2* (Bruker, 2012 ▸) and *SAINT* (Bruker, 2012 ▸), *SHELXT* (Sheldrick, 2015*a* ▸), *SHELXL2014* (Sheldrick, 2015*b* ▸), *DIAMOND* (Brandenburg, 1999 ▸).

## supplementary crystallographic information

### Crystal data

**Table:**

(C_11_H_18_N_4_)[Mo_8_O_26_]	*Z* = 1
*M**_r_* = 1596.11	*F*(000) = 768
Triclinic, *P*1	*D*_x_ = 2.532 Mg m^−^^3^
*a* = 8.6394 (10) Å	Mo *K*α radiation, λ = 0.71073 Å
*b* = 12.0694 (13) Å	Cell parameters from 9149 reflections
*c* = 12.2249 (14) Å	θ = 2.5–29.0°
α = 113.343 (3)°	µ = 2.42 mm^−^^1^
β = 110.629 (4)°	*T* = 180 K
γ = 96.540 (4)°	Plate, colourless
*V* = 1046.6 (2) Å^3^	0.28 × 0.18 × 0.15 mm

### Data collection

**Table:**

Bruker D8 QUEST diffractometer	5605 independent reflections
Radiation source: Sealed tube	4669 reflections with *I* > 2σ(*I*)
Multi-layer X-ray mirror monochromator	*R*_int_ = 0.032
Detector resolution: 10.4167 pixels mm^-1^	θ_max_ = 29.1°, θ_min_ = 3.6°
ω / φ scans	*h* = −11→11
Absorption correction: multi-scan (*SADABS*; Bruker, 2001)	*k* = −16→15
*T*_min_ = 0.595, *T*_max_ = 0.746	*l* = −16→16
58761 measured reflections	

### Refinement

**Table:**

Refinement on *F*^2^	4 restraints
Least-squares matrix: full	Hydrogen site location: mixed
*R*[*F*^2^ > 2σ(*F*^2^)] = 0.021	H atoms treated by a mixture of independent and constrained refinement
*wR*(*F*^2^) = 0.045	*w* = 1/[σ^2^(*F*_o_^2^) + (0.0188*P*)^2^ + 0.8837*P*] where *P* = (*F*_o_^2^ + 2*F*_c_^2^)/3
*S* = 1.05	(Δ/σ)_max_ = 0.002
5605 reflections	Δρ_max_ = 0.45 e Å^−^^3^
305 parameters	Δρ_min_ = −0.38 e Å^−^^3^

### Special details

**Table:**

Geometry. All e.s.d.'s (except the e.s.d. in the dihedral angle between two l.s. planes) are estimated using the full covariance matrix. The cell e.s.d.'s are taken into account individually in the estimation of e.s.d.'s in distances, angles and torsion angles; correlations between e.s.d.'s in cell parameters are only used when they are defined by crystal symmetry. An approximate (isotropic) treatment of cell e.s.d.'s is used for estimating e.s.d.'s involving l.s. planes.

### Fractional atomic coordinates and isotropic or equivalent isotropic displacement parameters (Å^2^)

**Table:**

	*x*	*y*	*z*	*U*_iso_*/*U*_eq_	
Mo1	−0.21482 (2)	0.51375 (2)	0.44384 (2)	0.01325 (5)	
Mo2	−0.03289 (2)	0.47766 (2)	0.25116 (2)	0.01588 (5)	
Mo3	0.09806 (2)	0.76466 (2)	0.66882 (2)	0.01539 (5)	
Mo4	0.29881 (2)	0.72827 (2)	0.48193 (2)	0.01568 (5)	
O1	0.03817 (17)	0.59486 (13)	0.47061 (13)	0.0144 (3)	
O2	0.20451 (18)	0.57180 (14)	0.31700 (14)	0.0173 (3)	
O3	0.5019 (2)	0.78869 (15)	0.50477 (16)	0.0241 (3)	
O4	0.30714 (18)	0.80224 (14)	0.65515 (14)	0.0181 (3)	
O5	0.1782 (2)	0.81272 (15)	0.42422 (15)	0.0231 (3)	
O6	0.1581 (2)	0.85684 (15)	0.83252 (15)	0.0250 (4)	
O7	−0.10203 (18)	0.63200 (13)	0.63336 (13)	0.0154 (3)	
O8	−0.0215 (2)	0.83770 (14)	0.59176 (15)	0.0221 (3)	
O9	−0.37025 (18)	0.41295 (14)	0.45016 (14)	0.0171 (3)	
O10	−0.32215 (19)	0.60161 (14)	0.38551 (14)	0.0194 (3)	
O11	−0.20542 (18)	0.39078 (13)	0.28676 (13)	0.0158 (3)	
O12	−0.0557 (2)	0.35530 (15)	0.10928 (15)	0.0250 (4)	
O13	−0.1414 (2)	0.57098 (15)	0.20309 (16)	0.0240 (3)	
N1	0.3125 (3)	0.17745 (19)	−0.33840 (19)	0.0245 (4)	
H1	0.327 (3)	0.200 (2)	−0.400 (2)	0.037*	
N2	0.1581 (3)	0.15545 (18)	−0.33513 (19)	0.0236 (4)	
H2	0.065 (2)	0.170 (3)	−0.391 (2)	0.035*	
N3	0.2863 (2)	0.27594 (18)	0.22445 (18)	0.0216 (4)	
H3	0.242 (3)	0.280 (2)	0.285 (2)	0.032*	
N4	0.3249 (2)	0.37686 (18)	0.20751 (19)	0.0222 (4)	
H4	0.302 (3)	0.4503 (16)	0.255 (2)	0.033*	
C1	0.6179 (3)	0.2024 (3)	−0.2101 (3)	0.0315 (6)	
H1A	0.6526	0.2767	−0.2201	0.047*	
H1B	0.6313	0.1287	−0.2749	0.047*	
H1C	0.6910	0.2175	−0.1205	0.047*	
C2	0.4340 (3)	0.1784 (2)	−0.2327 (2)	0.0208 (5)	
C3	0.3525 (3)	0.1549 (2)	−0.1608 (2)	0.0167 (4)	
C4	0.1777 (3)	0.1416 (2)	−0.2286 (2)	0.0190 (4)	
C5	0.0296 (3)	0.1179 (2)	−0.1979 (2)	0.0279 (5)	
H5A	−0.0785	0.0794	−0.2794	0.042*	
H5B	0.0260	0.1980	−0.1351	0.042*	
H5C	0.0434	0.0607	−0.1587	0.042*	
C6	0.4344 (3)	0.1463 (2)	−0.0344 (2)	0.0188 (4)	
H6A	0.5618	0.1776	0.0017	0.023*	
H6B	0.4039	0.0565	−0.0549	0.023*	
C7	0.2913 (3)	0.0554 (2)	0.1437 (2)	0.0265 (5)	
H7A	0.2436	0.0594	0.2065	0.040*	
H7B	0.4020	0.0367	0.1703	0.040*	
H7C	0.2101	−0.0113	0.0548	0.040*	
C8	0.3187 (3)	0.1786 (2)	0.1426 (2)	0.0181 (4)	
C9	0.3792 (3)	0.2204 (2)	0.0700 (2)	0.0167 (4)	
C10	0.3814 (3)	0.3458 (2)	0.1134 (2)	0.0194 (4)	
C11	0.4314 (3)	0.4388 (2)	0.0725 (2)	0.0280 (5)	
H11A	0.4545	0.5243	0.1415	0.042*	
H11B	0.3371	0.4233	−0.0105	0.042*	
H11C	0.5359	0.4305	0.0594	0.042*	

### Atomic displacement parameters (Å^2^)

**Table:**

	*U*^11^	*U*^22^	*U*^33^	*U*^12^	*U*^13^	*U*^23^
Mo1	0.01265 (9)	0.01485 (9)	0.01065 (9)	0.00461 (7)	0.00402 (7)	0.00530 (7)
Mo2	0.01593 (9)	0.01973 (10)	0.01075 (9)	0.00480 (7)	0.00481 (7)	0.00702 (7)
Mo3	0.01680 (9)	0.01357 (9)	0.01255 (9)	0.00375 (7)	0.00645 (7)	0.00330 (7)
Mo4	0.01521 (9)	0.01665 (10)	0.01562 (9)	0.00504 (7)	0.00687 (7)	0.00775 (7)
O1	0.0138 (7)	0.0161 (7)	0.0114 (7)	0.0040 (6)	0.0047 (6)	0.0055 (6)
O2	0.0176 (7)	0.0210 (8)	0.0145 (7)	0.0060 (6)	0.0081 (6)	0.0083 (6)
O3	0.0201 (8)	0.0265 (9)	0.0267 (9)	0.0054 (7)	0.0115 (7)	0.0127 (7)
O4	0.0158 (7)	0.0176 (8)	0.0151 (7)	0.0020 (6)	0.0045 (6)	0.0050 (6)
O5	0.0253 (8)	0.0244 (9)	0.0235 (8)	0.0106 (7)	0.0105 (7)	0.0139 (7)
O6	0.0301 (9)	0.0218 (8)	0.0158 (8)	0.0046 (7)	0.0092 (7)	0.0036 (7)
O7	0.0154 (7)	0.0162 (7)	0.0121 (7)	0.0039 (6)	0.0061 (6)	0.0044 (6)
O8	0.0229 (8)	0.0200 (8)	0.0229 (8)	0.0075 (7)	0.0098 (7)	0.0094 (7)
O9	0.0145 (7)	0.0192 (8)	0.0156 (7)	0.0049 (6)	0.0051 (6)	0.0075 (6)
O10	0.0193 (8)	0.0207 (8)	0.0182 (8)	0.0082 (6)	0.0067 (6)	0.0098 (6)
O11	0.0147 (7)	0.0175 (8)	0.0112 (7)	0.0039 (6)	0.0038 (6)	0.0049 (6)
O12	0.0259 (9)	0.0284 (9)	0.0159 (8)	0.0052 (7)	0.0085 (7)	0.0072 (7)
O13	0.0219 (8)	0.0309 (9)	0.0236 (8)	0.0101 (7)	0.0081 (7)	0.0177 (7)
N1	0.0309 (11)	0.0266 (11)	0.0215 (10)	0.0096 (9)	0.0126 (9)	0.0151 (9)
N2	0.0243 (10)	0.0250 (11)	0.0203 (10)	0.0107 (8)	0.0055 (8)	0.0123 (8)
N3	0.0214 (10)	0.0241 (10)	0.0181 (9)	0.0042 (8)	0.0127 (8)	0.0059 (8)
N4	0.0198 (10)	0.0206 (10)	0.0201 (10)	0.0071 (8)	0.0083 (8)	0.0042 (8)
C1	0.0293 (13)	0.0458 (16)	0.0349 (14)	0.0136 (12)	0.0208 (12)	0.0265 (13)
C2	0.0255 (12)	0.0210 (12)	0.0194 (11)	0.0087 (9)	0.0107 (9)	0.0115 (9)
C3	0.0201 (11)	0.0164 (11)	0.0150 (10)	0.0071 (8)	0.0089 (9)	0.0069 (8)
C4	0.0215 (11)	0.0148 (11)	0.0199 (11)	0.0074 (9)	0.0084 (9)	0.0074 (9)
C5	0.0215 (12)	0.0335 (14)	0.0304 (13)	0.0101 (10)	0.0128 (10)	0.0148 (11)
C6	0.0214 (11)	0.0247 (12)	0.0178 (11)	0.0103 (9)	0.0121 (9)	0.0127 (9)
C7	0.0346 (14)	0.0221 (12)	0.0201 (12)	0.0007 (10)	0.0132 (10)	0.0084 (10)
C8	0.0157 (10)	0.0216 (11)	0.0131 (10)	0.0028 (8)	0.0058 (8)	0.0057 (9)
C9	0.0132 (10)	0.0215 (11)	0.0136 (10)	0.0041 (8)	0.0049 (8)	0.0078 (9)
C10	0.0143 (10)	0.0237 (12)	0.0156 (10)	0.0051 (8)	0.0029 (8)	0.0083 (9)
C11	0.0323 (13)	0.0220 (12)	0.0236 (12)	0.0038 (10)	0.0048 (10)	0.0125 (10)

### Geometric parameters (Å, º)

**Table:**

Mo1—O10	1.6883 (14)	N2—C4	1.332 (3)
Mo1—O9	1.7506 (15)	N2—H2	0.935 (10)
Mo1—O11	1.9431 (14)	N3—N4	1.339 (3)
Mo1—O7	1.9561 (14)	N3—C8	1.342 (3)
Mo1—O1	2.1441 (14)	N3—H3	0.937 (10)
Mo1—O1^i^	2.3577 (14)	N4—C10	1.341 (3)
Mo1—Mo2	3.1874 (4)	N4—H4	0.937 (10)
Mo1—Mo3	3.2153 (4)	C1—C2	1.485 (3)
Mo2—O13	1.6939 (15)	C1—H1A	0.9800
Mo2—O12	1.7005 (16)	C1—H1B	0.9800
Mo2—O2	1.9304 (15)	C1—H1C	0.9800
Mo2—O11	1.9933 (15)	C2—C3	1.391 (3)
Mo2—O1	2.2824 (14)	C3—C4	1.397 (3)
Mo2—O7^i^	2.4033 (14)	C3—C6	1.508 (3)
Mo3—O6	1.6984 (15)	C4—C5	1.484 (3)
Mo3—O8	1.7063 (15)	C5—H5A	0.9800
Mo3—O4	1.8928 (15)	C5—H5B	0.9800
Mo3—O7	2.0078 (15)	C5—H5C	0.9800
Mo3—O1	2.2975 (14)	C6—C9	1.509 (3)
Mo3—O11^i^	2.3502 (14)	C6—H6A	0.9900
Mo4—O3	1.6999 (15)	C6—H6B	0.9900
Mo4—O5	1.7077 (15)	C7—C8	1.485 (3)
Mo4—O4	1.9147 (15)	C7—H7A	0.9800
Mo4—O2	1.9416 (15)	C7—H7B	0.9800
Mo4—O9^i^	2.2304 (15)	C7—H7C	0.9800
O1—Mo1^i^	2.3577 (14)	C8—C9	1.393 (3)
O7—Mo2^i^	2.4033 (14)	C9—C10	1.386 (3)
O9—Mo4^i^	2.2304 (15)	C10—C11	1.477 (3)
O11—Mo3^i^	2.3502 (14)	C11—H11A	0.9800
N1—C2	1.342 (3)	C11—H11B	0.9800
N1—N2	1.347 (3)	C11—H11C	0.9800
N1—H1	0.935 (10)		
			
O10—Mo1—O9	104.79 (7)	O4—Mo4—O9^i^	78.01 (6)
O10—Mo1—O11	101.71 (7)	O2—Mo4—O9^i^	77.29 (6)
O9—Mo1—O11	97.75 (6)	Mo1—O1—Mo2	92.07 (5)
O10—Mo1—O7	100.07 (7)	Mo1—O1—Mo3	92.69 (5)
O9—Mo1—O7	96.20 (6)	Mo2—O1—Mo3	160.42 (7)
O11—Mo1—O7	150.11 (6)	Mo1—O1—Mo1^i^	105.30 (6)
O10—Mo1—O1	99.34 (7)	Mo2—O1—Mo1^i^	99.34 (5)
O9—Mo1—O1	155.85 (6)	Mo3—O1—Mo1^i^	97.70 (5)
O11—Mo1—O1	78.04 (6)	Mo2—O2—Mo4	116.88 (7)
O7—Mo1—O1	78.43 (6)	Mo3—O4—Mo4	117.46 (7)
O10—Mo1—O1^i^	174.00 (6)	Mo1—O7—Mo3	108.41 (7)
O9—Mo1—O1^i^	81.15 (6)	Mo1—O7—Mo2^i^	108.04 (6)
O11—Mo1—O1^i^	78.01 (5)	Mo3—O7—Mo2^i^	103.79 (6)
O7—Mo1—O1^i^	78.19 (5)	Mo1—O9—Mo4^i^	120.16 (7)
O1—Mo1—O1^i^	74.70 (6)	Mo1—O11—Mo2	108.13 (7)
O10—Mo1—Mo2	89.83 (5)	Mo1—O11—Mo3^i^	109.11 (6)
O9—Mo1—Mo2	134.21 (5)	Mo2—O11—Mo3^i^	106.18 (6)
O11—Mo1—Mo2	36.46 (4)	C2—N1—N2	109.03 (18)
O7—Mo1—Mo2	124.12 (4)	C2—N1—H1	128.8 (17)
O1—Mo1—Mo2	45.69 (4)	N2—N1—H1	121.5 (17)
O1^i^—Mo1—Mo2	86.48 (3)	C4—N2—N1	109.52 (18)
O10—Mo1—Mo3	89.84 (5)	C4—N2—H2	129.5 (17)
O9—Mo1—Mo3	132.50 (5)	N1—N2—H2	119.3 (17)
O11—Mo1—Mo3	123.58 (4)	N4—N3—C8	109.53 (18)
O7—Mo1—Mo3	36.33 (4)	N4—N3—H3	120.5 (16)
O1—Mo1—Mo3	45.54 (4)	C8—N3—H3	129.9 (16)
O1^i^—Mo1—Mo3	85.40 (4)	N3—N4—C10	109.06 (18)
Mo2—Mo1—Mo3	89.640 (10)	N3—N4—H4	118.4 (16)
O13—Mo2—O12	105.61 (8)	C10—N4—H4	132.4 (16)
O13—Mo2—O2	101.67 (7)	C2—C1—H1A	109.5
O12—Mo2—O2	99.58 (7)	C2—C1—H1B	109.5
O13—Mo2—O11	99.61 (7)	H1A—C1—H1B	109.5
O12—Mo2—O11	99.06 (7)	C2—C1—H1C	109.5
O2—Mo2—O11	146.63 (6)	H1A—C1—H1C	109.5
O13—Mo2—O1	95.07 (7)	H1B—C1—H1C	109.5
O12—Mo2—O1	159.06 (7)	N1—C2—C3	107.6 (2)
O2—Mo2—O1	78.95 (5)	N1—C2—C1	120.7 (2)
O11—Mo2—O1	73.82 (5)	C3—C2—C1	131.8 (2)
O13—Mo2—O7^i^	165.12 (6)	C2—C3—C4	106.25 (19)
O12—Mo2—O7^i^	87.62 (6)	C2—C3—C6	127.50 (19)
O2—Mo2—O7^i^	82.35 (6)	C4—C3—C6	126.25 (19)
O11—Mo2—O7^i^	71.04 (5)	N2—C4—C3	107.61 (19)
O1—Mo2—O7^i^	71.46 (5)	N2—C4—C5	121.7 (2)
O13—Mo2—Mo1	86.49 (5)	C3—C4—C5	130.7 (2)
O12—Mo2—Mo1	134.42 (6)	C4—C5—H5A	109.5
O2—Mo2—Mo1	121.20 (4)	C4—C5—H5B	109.5
O11—Mo2—Mo1	35.40 (4)	H5A—C5—H5B	109.5
O1—Mo2—Mo1	42.24 (4)	C4—C5—H5C	109.5
O7^i^—Mo2—Mo1	79.26 (3)	H5A—C5—H5C	109.5
O6—Mo3—O8	104.89 (8)	H5B—C5—H5C	109.5
O6—Mo3—O4	102.17 (7)	C3—C6—C9	113.56 (17)
O8—Mo3—O4	101.97 (7)	C3—C6—H6A	108.9
O6—Mo3—O7	97.97 (7)	C9—C6—H6A	108.9
O8—Mo3—O7	96.88 (7)	C3—C6—H6B	108.9
O4—Mo3—O7	147.68 (6)	C9—C6—H6B	108.9
O6—Mo3—O1	161.95 (7)	H6A—C6—H6B	107.7
O8—Mo3—O1	92.20 (6)	C8—C7—H7A	109.5
O4—Mo3—O1	79.39 (6)	C8—C7—H7B	109.5
O7—Mo3—O1	73.84 (5)	H7A—C7—H7B	109.5
O6—Mo3—O11^i^	90.45 (7)	C8—C7—H7C	109.5
O8—Mo3—O11^i^	162.37 (6)	H7A—C7—H7C	109.5
O4—Mo3—O11^i^	82.78 (6)	H7B—C7—H7C	109.5
O7—Mo3—O11^i^	71.98 (5)	N3—C8—C9	107.23 (19)
O1—Mo3—O11^i^	71.81 (5)	N3—C8—C7	120.65 (19)
O6—Mo3—Mo1	133.10 (6)	C9—C8—C7	132.1 (2)
O8—Mo3—Mo1	84.13 (5)	C10—C9—C8	106.44 (19)
O4—Mo3—Mo1	121.16 (4)	C10—C9—C6	126.00 (19)
O7—Mo3—Mo1	35.26 (4)	C8—C9—C6	127.6 (2)
O1—Mo3—Mo1	41.77 (3)	N4—C10—C9	107.7 (2)
O11^i^—Mo3—Mo1	78.97 (4)	N4—C10—C11	121.0 (2)
O3—Mo4—O5	105.36 (8)	C9—C10—C11	131.3 (2)
O3—Mo4—O4	105.00 (7)	C10—C11—H11A	109.5
O5—Mo4—O4	96.53 (7)	C10—C11—H11B	109.5
O3—Mo4—O2	104.14 (7)	H11A—C11—H11B	109.5
O5—Mo4—O2	97.61 (7)	C10—C11—H11C	109.5
O4—Mo4—O2	142.75 (6)	H11A—C11—H11C	109.5
O3—Mo4—O9^i^	94.00 (6)	H11B—C11—H11C	109.5
O5—Mo4—O9^i^	160.64 (7)		
			
O6—Mo3—O4—Mo4	−175.31 (8)	N1—N2—C4—C5	179.2 (2)
O8—Mo3—O4—Mo4	−66.98 (9)	C2—C3—C4—N2	0.6 (2)
O7—Mo3—O4—Mo4	57.37 (15)	C6—C3—C4—N2	−179.9 (2)
O1—Mo3—O4—Mo4	23.03 (8)	C2—C3—C4—C5	−178.8 (2)
O11^i^—Mo3—O4—Mo4	95.79 (8)	C6—C3—C4—C5	0.7 (4)
Mo1—Mo3—O4—Mo4	23.37 (10)	C2—C3—C6—C9	133.1 (2)
O10—Mo1—O9—Mo4^i^	178.98 (8)	C4—C3—C6—C9	−46.3 (3)
O11—Mo1—O9—Mo4^i^	74.63 (8)	N4—N3—C8—C9	−0.5 (2)
O7—Mo1—O9—Mo4^i^	−78.87 (8)	N4—N3—C8—C7	178.4 (2)
O1—Mo1—O9—Mo4^i^	−3.4 (2)	N3—C8—C9—C10	0.3 (2)
O1^i^—Mo1—O9—Mo4^i^	−1.88 (7)	C7—C8—C9—C10	−178.5 (2)
Mo2—Mo1—O9—Mo4^i^	74.35 (9)	N3—C8—C9—C6	179.6 (2)
Mo3—Mo1—O9—Mo4^i^	−77.24 (9)	C7—C8—C9—C6	0.7 (4)
C2—N1—N2—C4	−0.2 (3)	C3—C6—C9—C10	−50.6 (3)
C8—N3—N4—C10	0.6 (2)	C3—C6—C9—C8	130.3 (2)
N2—N1—C2—C3	0.6 (3)	N3—N4—C10—C9	−0.4 (2)
N2—N1—C2—C1	−179.2 (2)	N3—N4—C10—C11	179.19 (19)
N1—C2—C3—C4	−0.7 (2)	C8—C9—C10—N4	0.0 (2)
C1—C2—C3—C4	179.0 (2)	C6—C9—C10—N4	−179.2 (2)
N1—C2—C3—C6	179.8 (2)	C8—C9—C10—C11	−179.5 (2)
C1—C2—C3—C6	−0.4 (4)	C6—C9—C10—C11	1.3 (4)
N1—N2—C4—C3	−0.2 (2)		

Symmetry code: (i) −*x*, −*y*+1, −*z*+1.

### Hydrogen-bond geometry (Å, º)

**Table:**

*D*—H···*A*	*D*—H	H···*A*	*D*···*A*	*D*—H···*A*
N1—H1···O10^ii^	0.94	2.34	2.941 (2)	122
N2—H2···O5^ii^	0.94	2.05	2.852 (2)	143
N2—H2···O8^ii^	0.94	2.31	2.977 (2)	127
N3—H3···O7^i^	0.94	1.97	2.759 (2)	141
N4—H4···O2	0.94	1.81	2.730 (2)	166

Symmetry codes: (i) −*x*, −*y*+1, −*z*+1; (ii) −*x*, −*y*+1, −*z*.
